# Cryopreservation of Human Adult Ventricular Tissue for the Preparation of Viable Myocardial Slices

**DOI:** 10.1002/cpz1.70068

**Published:** 2024-12-03

**Authors:** Alessandra M. Lodrini, Esmee J. Groeneveld, Meindert Palmen, Jesper Hjortnaes, Anke M. Smits, Marie‐José Goumans

**Affiliations:** ^1^ Department of Cell and Chemical Biology Leiden University Medical Center Leiden Netherlands; ^2^ Department of Cardiothoracic Surgery Leiden University Medical Center Leiden Netherlands

**Keywords:** cryopreservation, human myocardium, living myocardial slices

## Abstract

Living myocardial slices (LMS) are ultrathin sections of adult myocardium that can be maintained in culture. These slices provide a unique platform for studying interactions between cardiomyocytes (CMs), other cardiac cell types, and the extracellular matrix while maintaining the cytoarchitecture and electrical phenotype of CMs over extended periods. Despite their advantages over other cardiac models, LMS have limitations, particularly their reliance on slice quality. The primary factor influencing the quality of the slices is the method used to process the cardiac tissue block. Current methods typically require immediate slice preparation following the excision of the tissue block, which restricts the timing of experiments. To address this limitation, we developed a simple procedure for cryopreserving human adult myocardium, allowing the preparation of LMS at a later stage. The protocol provides a list of required equipment and reagents, as well as a detailed description of the methodology for processing the myocardium and slice preparation. We present typical results demonstrating that cryopreserved human cardiac tissue retains biomass and structural integrity comparable to freshly obtained myocardium. Furthermore, we assessed the LMS derived from both fresh and cryopreserved samples. Histological analysis confirmed the preservation of viability, normal cytomorphology, and gap junctions between CMs in all LMS after 24 h and up to 5 days of culture in the absence of electrical stimulation. Cryopreservation extends the interval between tissue collection and LMS preparation, facilitating longer‐term and more complex experiments. Further research into the impact of cryopreservation on various cardiac cell types will promote better donor organ management and efficient banking of cardiac samples from a multitude of donors and disease states. © 2024 The Author(s). Current Protocols published by Wiley Periodicals LLC.

**Basic Protocol 1**: Preparation and preservation of human adult myocardium

**Basic Protocol 2**: Preparation of adult living myocardial slices from cryopreserved blocks

## INTRODUCTION

Living myocardial slices (LMS) are thin (100‐400 µm) sections of living myocardium that can be maintained under culture conditions. The thin nature of LMS allows the diffusion of oxygen and nutrients from the culture medium directly into the tissue.

The first publications on myocardial slices date back to 1933 (Pincus, 1933) when thin preparations of rat ventricular tissue were used to test the effect of drugs on oxygen consumption of different tissues. They resurfaced in 1990 (Brunashev et al., 1990) when they were applied in electrophysiological research for the first time. Later, LMS became of interest to the scientific community after additional structural and electrophysiological characteristics of rat myocardium were unveiled, offering a new model to study electrical impulse propagation (Bursac et al., 2003; Pillekamp et al., 2006; Watson et al., 2019; Fahy and Wowk, 2015; Amini and Benson, 2023; Hall and Hausenloy, 2016; Horn and Trafford, 2016).

Before 2011, LMS could be cultured for very short periods (<24 h). To prolong this period and preserve tissue structure, liquid–air interface culture systems with oxygenation, nutrient support, and electrical stimulation were developed (Brandenburger et al., 2012; Kang et al., 2016; Ou et al., 2019; Watson et al., 2017), allowing culture up to 28 days. Mechanical loading was subsequently introduced and several groups have shown that human LMS can be maintained for up to 4 months when cultured under auxotonic loading, 0.2 Hz stimulation, and media agitation in a bioengineered device (Fischer et al., 2019; Miller et al., 2022; Pitoulis et al., 2022; Qiao et al., 2019; Watson et al., 2019). However, under these conditions, LMS still undergo a certain degree of remodeling, as reflected by gene ontology analysis and RNAseq data (Fischer et al., 2019; Miller et al., 2022; Ou et al., 2020; Pitoulis et al., 2022). Therefore, there is a continuous effort to further optimize the physiological long‐term cultures of LMS.

LMS can be prepared from small and large mammalian hearts, including humans. Human tissue is usually obtained from donor organs or patients undergoing open‐heart surgery, such as atrial appendage amputations, septal myectomies, left‐ventricular assist device implantations, and cardiac transplantations.

As multicellular 3D culture models, LMS offer a novel opportunity to investigate how adult cardiomyocytes (CMs) interact with other cardiac cell types and with the extracellular matrix (ECM) while providing long‐term preservation of CM cytoarchitecture and electrical phenotype (Qiao et al., 2019). Additionally, they provide a new tool in drug discovery by allowing the testing of chronic treatments as opposed to isolated adult CMs, which are typically limited to acute time points.

Despite their benefits over other cardiac preparations, LMS also have their limitations. The biggest determinant of slice quality is the method by which the cardiac sample is handled. Current methods depend on the preparation of LMS directly after excision of the cardiac sample from the heart, thereby limiting the timing of the experiments and complicating the inclusion of biological replicates within one experiment. Cold storage could extend the time interval from sample collection to slice preparation, facilitating the distribution of LMS obtained from scarcely available human organs and providing a regular inflow of material that is needed to advance research.

Cryopreservation of cells and tissues is a common practice in biomedical research. Methods for slow and rapid freezing of tissue slices have been previously described for human lung slices (Bull, Connors et al., 2000; Patel et al., 2023), rat and human liver and kidney slices (de Graaf et al., 2007; Fahy et al., 2013), and rat heart slices (Bull, Reid et al., 2000). As cryopreservation will enable the use of LMS in myocardial research, we developed a protocol to preserve the human myocardium for subsequent LMS preparation.

*NOTE*: All protocols involving animals must be reviewed and approved by the appropriate Animal Care and Use Committee and must follow regulations for the care and use of laboratory animals. Appropriate informed consent is necessary for obtaining and use of human study material.

## Basic Protocol 1 PREPARATION AND PRESERVATION OF HUMAN ADULT MYOCARDIUM

This protocol outlines a straightforward method for preparing human adult ventricular myocardium for cryopreservation.

### Materials

Fresh human cardiac tissue (at least ∼1 cm^3^)
Washing solution (See recipe)
Cryopreservation medium (Gibco CTS Synth‐a‐Freeze Medium; Thermo Fisher, cat. no. A1371301)
Liquid nitrogen

Specimen container (e.g., sterile 50‐mL tube with 10 mL of culture medium or slicing solution)
15‐cm petri dish
Disposable sterile scalpel
Noyes spring scissors
Dumont tweezers
Cryovials (Nunc Biobanking and Cell Culture Cryogenic Tubes; Thermo Scientific, cat. no. 366656)
Laminar flow cabinet with UV light
Cell‐freezing container (Nalgene Mr Frosty; Sigma‐Aldrich, cat. no. C1562)
−80°C freezer

*NOTE*: All experiments must comply with institutional and national regulations. This study was conducted in accordance with the Ethical Principles of the Declaration of Helsinki 2013 and according to the Dutch regulation for the responsible use of human tissues for medical research purposes.

*NOTE*: Depending on the experiment, ensure adequate cleaning and/or sterilization of the equipment.

*NOTE*: After excision from the patient, the myocardial tissue should be stored in a physiological solution (e.g., Tyrode solution) or culture medium (e.g., Medium‐199) at 4°C and processed as quickly as possible to ensure viability.

### Procedure

**Figure 1:**
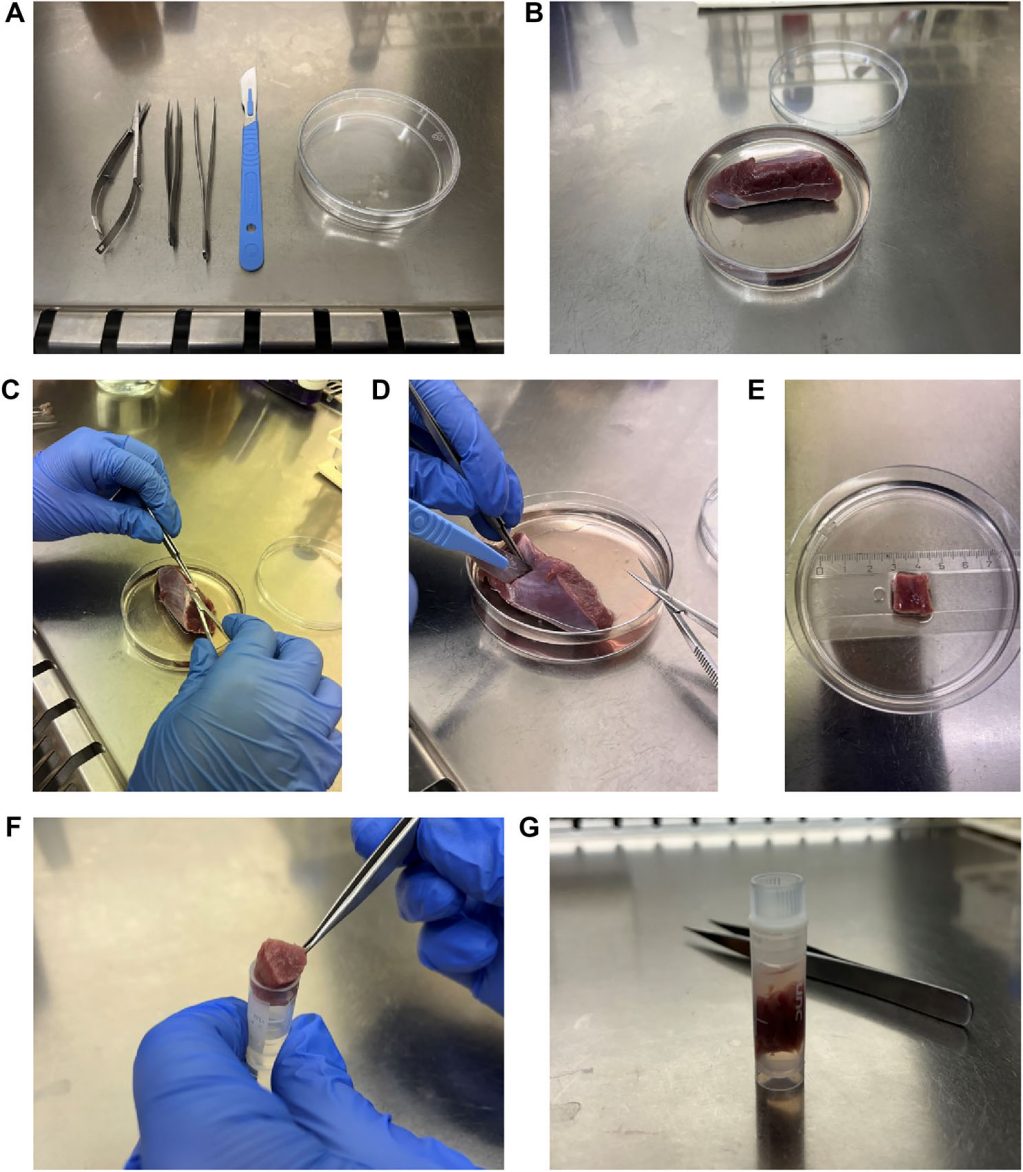
Preparation and preservation of human adult myocardium. **(A)** Suggested setup for the dissection area. **(B)** Washing the tissue using the washing solution. **(C)** Removing any fat or other non‐myocardial tissue using scissors. **(D)** Cutting the tissue starting from the endocardial side. **(E)** Preparation of small blocks. **(F)** Insertion of the block into a cryovial containing cryopreservation medium. **(G)** Immersion of the block in the cryopreservation medium.

1 Remove the tissue from the specimen container and place it in a 15‐cm petri dish.
2 Wash the tissue using cold (4°C) washing solution to remove any blood (Fig. 1B).

3 Use a scalpel or scissors to remove any fat or other non‐myocardial tissue if present (Fig. 1C).
4 Identify the endocardial and epicardial sides of the tissue by looking for a smooth, shiny surface covered by a thin layer of fat and blood vessels.
5 Using a scalpel or scissors, prepare small blocks (up to 1 cm^3^) starting from the epicardial or endocardial side (Fig. 1D and E). This helps identify the alignment of the myocardial fibers within the tissue.
6 Lightly blot the surface of the blocks with tissue paper to remove excess solution from its surface.
7 Gently place each block in a cryovial containing ∼1 mL of cryopreservation medium (Fig. 1F and G). Ensure that the block is completely immersed in the cryopreservation medium.
8 Freeze appropriately.
  NOTE: We tested both rapid freezing using liquid nitrogen and slow freezing overnight using a cell‐freezing container. We did not find significant morphological differences between these two freezing methods; however, the most appropriate method of freezing may depend on the research question.
9 Store at −80°C.

## Basic Protocol 2 PREPARATION OF ADULT LIVING MYOCARDIAL SLICES FROM CRYOPRESERVED BLOCKS

This protocol illustrates how to prepare LMS from cryopreserved blocks of tissue prepared using the previous protocol.

### Materials

Cryopreserved blocks from Basic Protocol 1
Slicing solution (See recipe)
4% agarose solution (low‐gelling‐temperature agarose; Sigma‐Aldrich, cat. no. A0701)
*NOTE*: To prepare the agarose solution, dissolve 4% low‐gelling‐temperature agarose in the washing solution (4 g in 100 mL). Heat and stir until the agarose has melted (∼80°C). Leave to cool at room temperature, and then store at 4°C.
Histoacryl surgical glue (Braun, cat. no. 1050052)
Culture medium complete with required nutrients (See recipe)
Tyrode solution (slicing solution without BDM)
Washing solution (See recipe)

15‐cm petri dish
Embedding mold (Peel‐A‐Way embedding molds; Sigma‐Aldrich, cat. no. E6032)
Disposable sterile scalpel
Straight flat‐tip tweezers
Vibrating microtome (Leica VT1200S)
*NOTE*: Alternative vibratomes can be used if they can be calibrated for the Z‐axis error (<1.0 µm) and advance at slow speeds.
Vibratome blades (Feather Double Edge Carbon Steel Blades; Ted Pella, cat. no. 121‐9)
Dumont tweezers
6‐well culture plates (Cellstar, GBO)
Laminar flow cabinet with UV light
0.4‐µm semiporous tissue culture inserts (Millipore, cat. no. PICM0RG50)
37°C and 5% CO_2_ incubator

### Procedure

**Figure 2:**
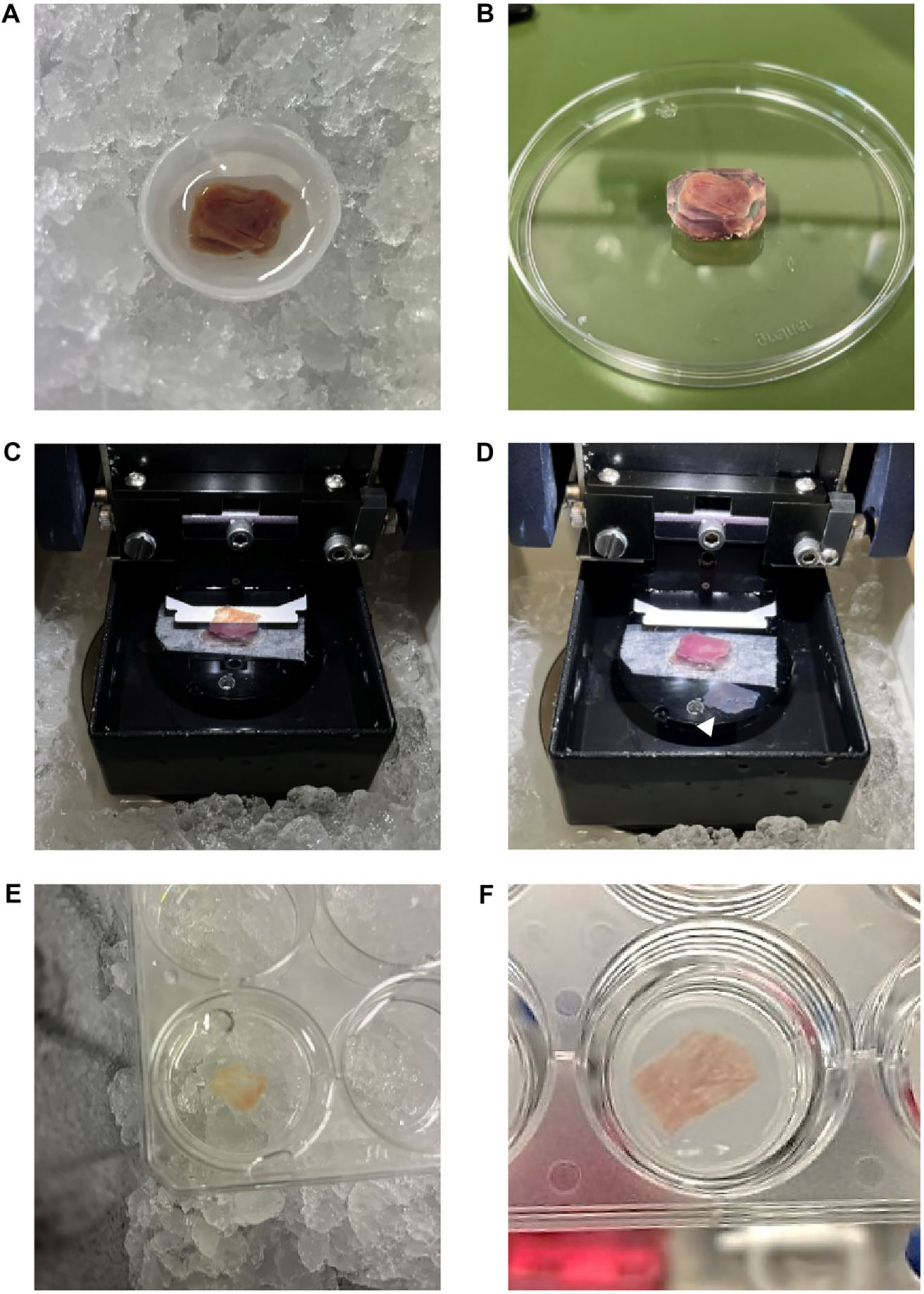
Preparation of adult living myocardial slices. **(A)** Embedding the tissue block in low‐gelling‐temperature agarose solution, avoiding air bubbles. **(B)** Cutting off excess agarose around the block. **(C)** Attachment of the specimen holder to the inner part of the vibratome and slicing. **(D)** Detachment of the LMS (white arrow) from the superior surface of the block. **(E)** Transfer of the LMS to a cold holding bath containing Tyrode solution. **(F)** Placing the LMS in the culture plate, flat on the surface of the tissue culture insert gauze.

1 Place the cryovial at 4°C until the tissue block is completely thawed.
2 Transfer the block from the cryovial to a 15‐cm petri dish.
3 Wash the block using cold slicing solution to remove any cryopreservation medium.
4 Heat the agarose solution until it melts and then let it cool to ∼37°C.
5 Lightly blot the block with tissue paper to remove excess solution from its surface and place it in an embedding mold.
6 Embed the block in agarose by pouring the cooled agarose into the embedding mold, trying to avoid air bubbles (Fig. 2A).
  NOTE: The tissue is embedded in agarose with a low melting temperature to stabilize the block and to guarantee uniformity between LMS. The agarose should be liquid for embedding, but the temperature should not exceed 37°C to avoid damaging the tissue.

7 Let the agarose solidify for 3 min by placing the mold in ice; then remove it from the mold and cut excess agarose around the block with a scalpel (Fig. 2B).
8 Apply a drop of Histoacryl glue to the specimen holder of the vibratome.
9 Gently pick up the embedded tissue by one of its lateral edges using flat‐tip tweezers. Position the embedded tissue with the epicardial/endocardial surface down on top of the Histoacryl glue drop.
10 Apply gentle pressure to ensure the tissue attaches.
  NOTE: The embedded tissue will start attaching to the holder immediately upon contact. It is not possible to remove the embedded tissue once stuck.
11 Rapidly transfer the specimen holder to the inner part of the vibratome bath containing the slicing solution.
12 Position the vibratome blade in the correct starting position. Advance the blade until it reaches the front edge of the tissue block. Modify the blade height to match the upper edge of the tissue block.
  NOTE: Before starting, ensure Z‐axis error < 1.0 µm, as described in the vibratome user manual. The blade should be set to vibrate at a frequency of 80 Hz and an amplitude of 2 mm, with an advancing speed of 0.04 mm/s. The section thickness should be 300 µm.
13 Start slicing (Fig. 2C).
14 As slices detach from the superior surface of the block, carefully transfer all the LMS to a culture plate containing cold (4°C) Tyrode solution using flat‐tip tweezers (Fig. 2D and E).
  NOTE: To ensure viability, LMS should not remain in the holding bath for more than 5 h.
15 To place the LMS in culture, wash them with the washing solution at room temperature in a sterile flow cabinet.
16 Gently separate the agarose from the tissue slice using a scalpel and/or tweezers.
17 Place the inserts in the culture plate and add 1 mL of culture medium to each well.
18 Carefully transfer the LMS to the wells using tweezers. Make sure they are flat on the surface of the tissue culture insert gauze (Fig. 2F).
19 Cover the plate and incubate at 37°C with 5% CO_2_. Replace the culture medium every 24‐48 h.
  NOTE: Penicillin‐streptomycin in the culture medium can be reduced to 1% after 24 h.

## REAGENTS AND SOLUTIONS

### Culture medium

Gibco Medium‐199 (Thermo Fisher Scientific, cat. no. 31150022)
ITS liquid media supplement, 0.001% (Sigma‐Aldrich, cat. no. I3146)
Gibco Penicillin‐Streptomycin, 3% (Thermo Fisher Scientific, cat. no. 15070063)

### Slicing solution

140 mM NaCl
6 mM KCl
1.8 mM CaCl_2_
1 mM MgCl_2_
10 mM HEPES
1 mM d‐glucose
30 mM 2,3‐butanedione monoxime (BDM)
Adjust pH to 7.4

### Washing solution

Gibco phosphate‐buffered saline with calcium and magnesium (Thermo Fisher Scientific, cat. no. 14040133)
Gibco Penicillin‐Streptomycin, 3% (Thermo Fisher Scientific, cat. no. 15070063)

### Critical Parameters/Troubleshooting

This protocol describes a simple procedure for the cryopreservation of human adult ventricular tissue for the preparation of LMS. By cryopreservation, living material is brought to a state in which biological processes come to a standstill. The challenge of designing cryopreservation protocols is to prevent damage during cooling and rewarming to physiological temperatures.

In this study, cardiac tissue was obtained from the surgical waste material of patients undergoing Morrow myectomy, left‐ventricular assist device implantation, or valve surgery. All tissue samples were collected with patient consent under appropriate ethical approval. Immediately following excision, the tissue was placed in a sterile specimen holder containing culture medium or slicing solution and maintained at 4°C. This interim cold storage was to minimize tissue degradation until the start of the cryopreservation protocol, with a maximum delay of 2 h between excision and freezing.

Critical steps in the protocol are the correct processing of the tissue into blocks for cryopreservation, as well as the choice of a suitable freezing method. Tissue blocks should be cut starting from the epicardial or endocardial side to identify the alignment of the myocardial fibers in subsequent steps. Indeed, only areas with myocardial fibers cut longitudinally and running parallel to each other should be used for structural and functional studies. The blocks should fit comfortably in a cryovial and be surrounded by the cryopreservation medium to protect the integrity of the tissue. The cryopreservation medium is essential to limit ice formation in the extracellular space and cell shrinkage in response to ice formation, thereby reducing damage and cell death (Fahy et al., 2013). After testing different cryopreservation media formulations, we settled on a commercial medium to obtain a freezing profile that is highly consistent and reproducible.

We tested both rapid freezing using liquid nitrogen and slow freezing using a cell‐freezing container with a −1°C/min freezing rate at −80°C. In our experiments, we did not observe any significant differences between the two methods of freezing in terms of apoptosis and morphological markers. However, the most appropriate method for other readouts may depend on the final use of the tissue. For example, if the tissue will be used for metabolic studies, rapid freezing may be more suitable to preserve metabolites, as previously reported for other tissues (Bull et al., 2000; de Graaf et al., 2007). However, morphological and functional studies may benefit from slow freezing to minimize the chance of intracellular ice formation and consequent structural damage (Bojic et al., 2021; Wang et al., 2007). Additionally, different cells within a complex tissue like the myocardium may vary in water permeability, raising the concern that the optimum cooling rate for one cell type of interest may differ from another (Fahy et al., 2013). The current protocol can be adapted by using a combination of slow controlled‐rate freezing and rapid freezing or even vitrification (Amini and Benson, 2023; Fahy and Wowk, 2015). This may require a more sophisticated programmable cell‐freezing apparatus that allows the selection of variable cooling rates for different stages of the freezing process.

The described protocol allows the cryopreservation of human adult ventricular tissue for several weeks (up to 4 weeks). Our results show that the viability and coupling of stromal cells and CMs are maintained, indicating the feasibility of preparing LMS after cryopreservation. Although we did not perform functional measurements, we did observe preserved intercalated disks and gap junctions, which may indicate that propagation of electrical impulses and nutrients throughout the tissue could be possible. Of note, we did not include BDM in the bath holding the LMS. While BDM effectively suppresses contraction and ATPase activity, it has been reported to have off‐target effects, such as inhibiting mitochondrial respiration by acting on the electron transport chain (Hall and Hausenloy, 2016). To mitigate potential adverse effects, we minimized the exposure of our slices to BDM, allowing for a longer recovery period before culturing.

Cold storage extends the time interval between sample collection and LMS preparation, allowing for more long‐term and complex experimental endpoints. Moreover, efficient banking of cryopreserved cardiac samples from various donors and disease states is critical for research progression and it will permit more extensive use of LMS in drug discovery and development. Additional research on the effects of cryopreservation on different cardiac cell types may lead to improvements in donor organ management by extending the donor pool and increasing the utilization rate.

### Understanding the Results

In this section, we present typical data that can be collected using human myocardial samples and LMS, comparing fresh and cryopreserved tissue.

#### Tissue integrity and structure

**Figure 3:**
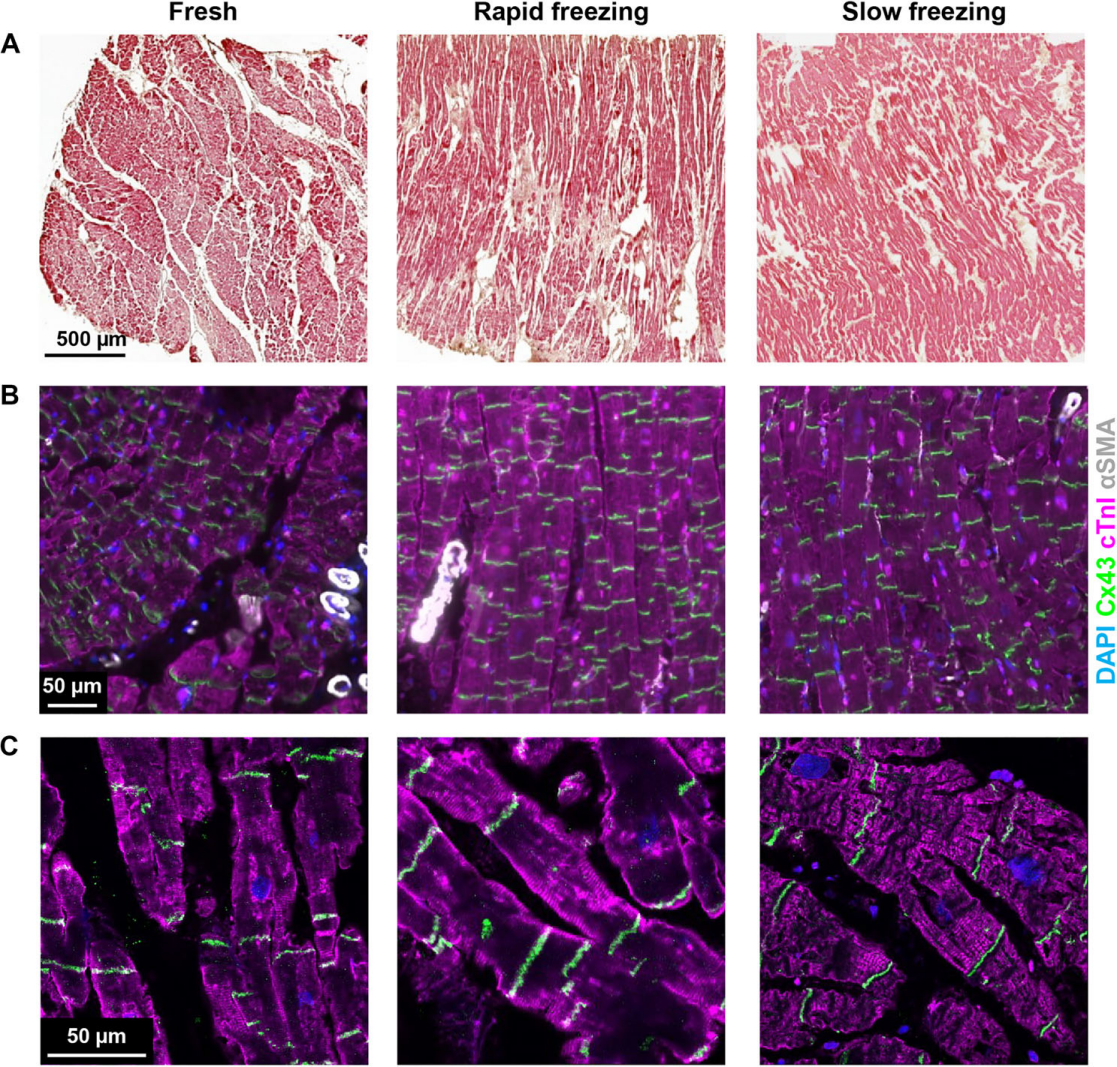
Tissue integrity and structure. **(A)** Representative images of hematoxylin and eosin staining of fresh and cryopreserved cardiac tissue. Myocardial fibers are shown in pink and the extracellular matrix is shown in brown. **(B)** Representative images of immunohistochemical analysis of fresh and cryopreserved cardiac tissue. **(C)** Closeups of cardiomyocytes from fresh and cryopreserved cardiac tissue using confocal imaging. Cardiomyocytes were stained with cardiac Troponin‐I antibody (cTnI, magenta), gap junctions between cells with connexin‐43 (Cx43, green), and stromal cell populations with alpha‐smooth muscle actin (αSMA, white). Counterstaining of nuclei was performed with DAPI (blue).

The structure of myocardial tissue can be studied using various techniques. We typically start by investigating the tissue integrity of the LMS using hematoxylin and eosin (H&E). H&E allows visualization of the overall myocardial fiber structure and orientation (pink) and the ECM (brown) (Fig. 3A). Following the described cryopreservation protocol, no loss of tissue integrity or sign of damage to cardiac fibers was observed when compared to fresh tissue. For structural and functional studies, only areas with myocardial fibers cut longitudinally and running parallel to each other (Fig. 3B) should be used.

We used immunohistochemistry (IHC) to study cellular composition, as previously described (Kruithof et al. 2024; Senesi et al. 2024). Briefly, fresh or cryopreserved (2‐4 weeks) tissue blocks were fixed by incubation with 4% PFA at room temperature for 4 h with gentle mechanical agitation and subsequently embedded in paraffin for sectioning. The sections were then deparaffinized and hydrated before staining. Additionally, at the end of the IHC protocol, we removed any autofluorescence by incubating the sections with 0.1% Sudan Black B (Sigma‐Aldrich, cat. no. 199664) in 70% ethanol for 30 min at room temperature. The Sudan Black B was then thoroughly washed with PBS before mounting the microscopy slides with ProLong Gold Antifade Mountant (Thermofisher, cat. no. P36930).

Different antibodies (Table 1) were applied to visualize different cell types by IHC and confocal microscopy. Cardiac troponin‐I (cTnI) antibody was used to identify sarcomeres, while gap junctions between cells were marked with connexin‐43 (Cx43). The microvasculature was characterized by CD‐31/PECAM‐1 (PECAM‐1) expression and the stromal cell populations were stained with alpha‐smooth muscle actin (αSMA).

**Table 1:** Primary Antibodies

Antibody	Dilution	Manufacturer	Cat. no.
Cardiac troponin‐I	1:1000	HyTest	4T21/2
Connexin‐43	1:500	Sigma‐Aldrich	C6219
CD‐31/PECAM‐1	1:200	R&D Systems	AF3628
α‐smooth muscle actin	1:200	Santa Cruz Biotechnology	SC‐32251
Type I Collagen	1:2000	Southern Biotech	1310‐01
Type III Collagen	1:2000	Southern Biotech	1330‐01

Myocardial fiber and cytoarchitecture appeared intact post‐cryopreservation (Fig. 3B and C). Cx43 expression was comparable and evenly distributed in cryopreserved myocardium compared to fresh tissue, indicating persistent coupling of CMs, which allows intracellular movement of ions and small metabolites.

**Figure 4:**
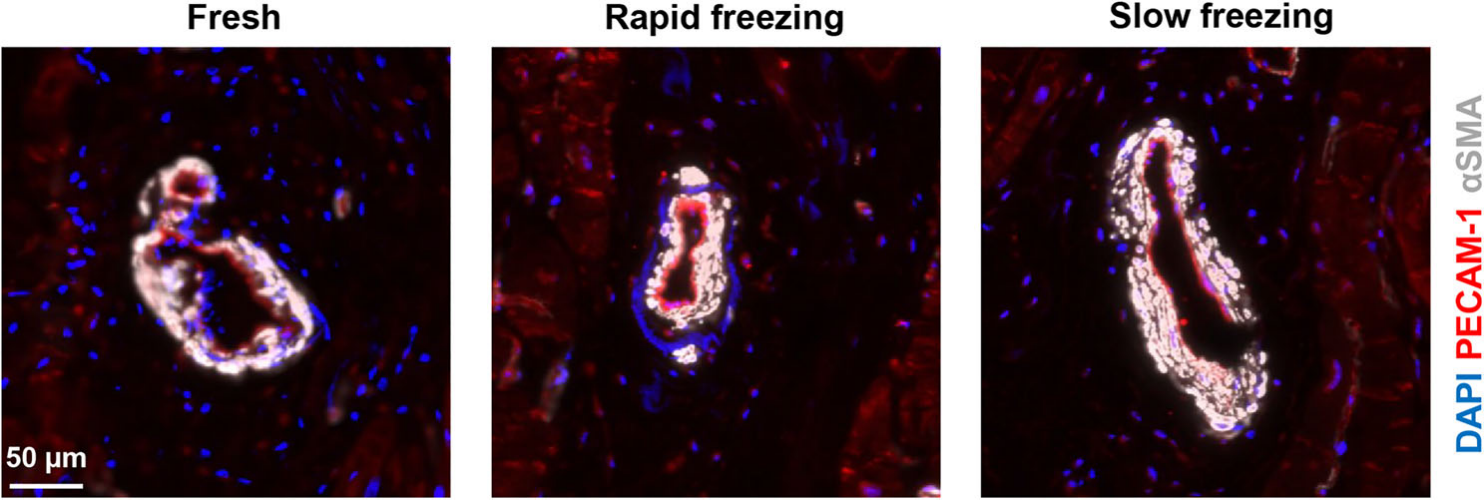
Vasculature. Representative images of immunohistochemical analysis of fresh and cryopreserved cardiac tissue. Endothelial cells were stained for CD31/PECAM‐1 (red) and smooth muscle cells with alpha‐smooth muscle actin (αSMA, white). Counterstaining of nuclei was performed with DAPI (blue).

We did not observe any vascular remodeling in cryopreserved myocardium compared with fresh tissue (Fig. 4). The vessel's lumen was easily recognized by PECAM‐1‐positive endothelial cells and the surrounding αSMA‐positive smooth muscle cells.

**Figure 5:**
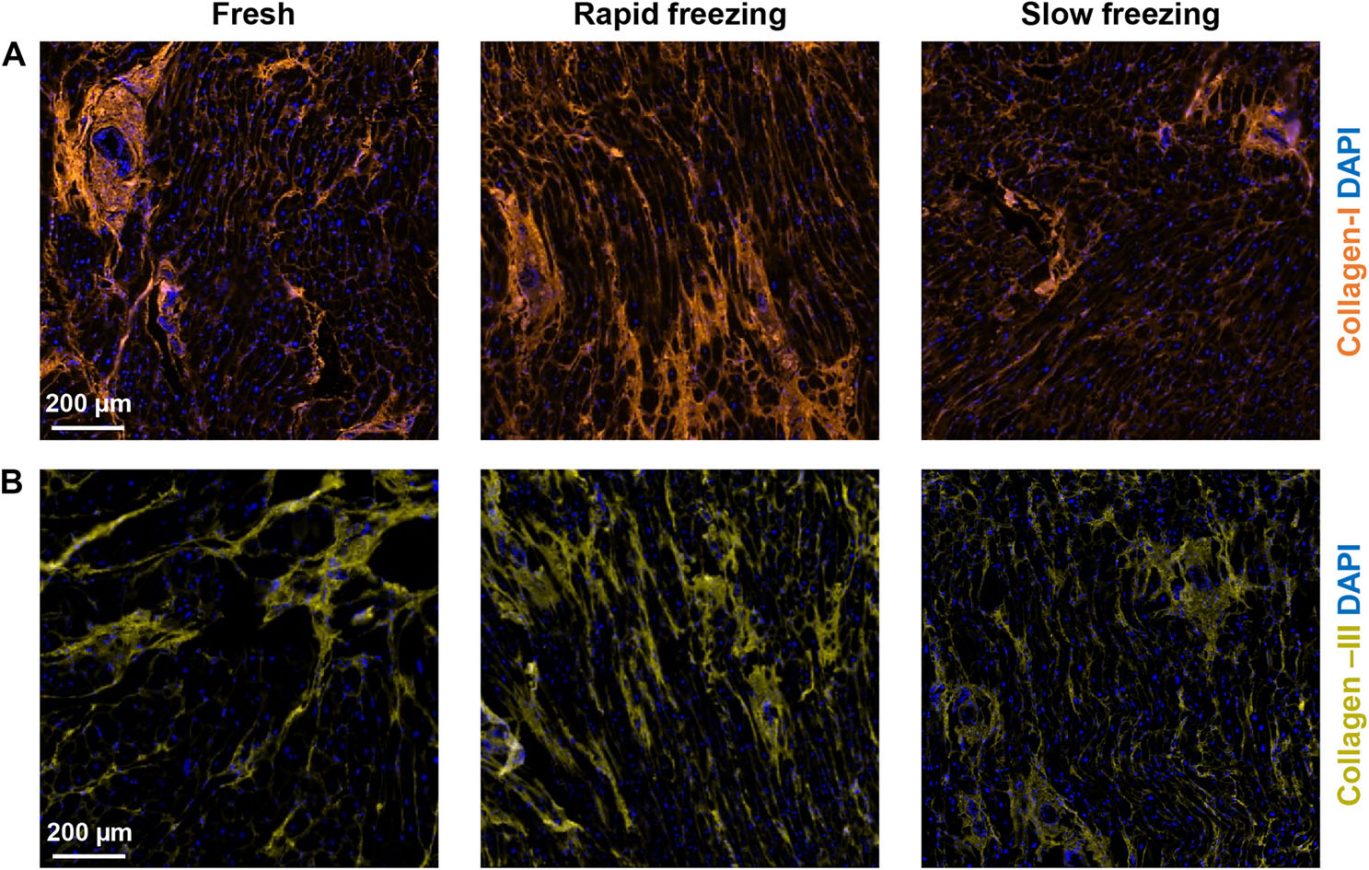
Extracellular matrix. Representative images of immunohistochemical analysis of fresh and cryopreserved cardiac tissue. Type I Collagen (Collagen‐I) is shown in orange **(A)** and Type III Collagen (Collagen‐III) is shown in yellow **(B)**. Counterstaining of nuclei was performed with DAPI (blue).

We assessed the effect of cryopreservation on the expression of Type I Collagen (Collagen‐I) and Type III Collagen (Collagen‐III), which together constitute 90% of the myocardial ECM (Horn and Trafford, 2016) (Fig. 5). We did not observe any significant difference in the expression and organization of Collagen‐I and Collagen‐III fibers in the cryopreserved samples compared to that in fresh tissue.

#### Apoptosis

**Figure 6:**
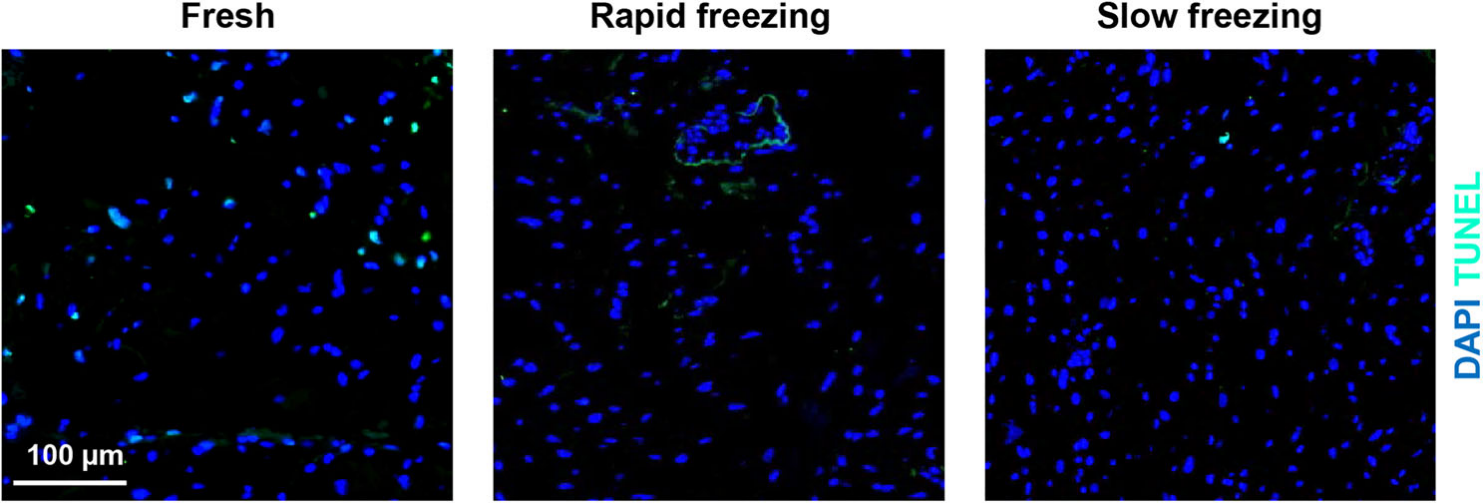
Viability assay. Apoptotic cells were labeled with TUNEL (cyan) (see Understanding the Results). Counterstaining of nuclei was performed with DAPI (blue).

The presence of apoptotic cells was assessed using the In Situ Cell Death Detection Kit, Fluorescein (Roche) as per the manufacturer's instructions. The kit allowed us to detect and quantify apoptosis at the single‐cell level, based on labeling of DNA strand breaks through TUNEL (terminal deoxynucleotidyl transferase dUTP nick end labeling) technology. Analysis was then performed by fluorescence microscopy. There was no significant difference in the number of TUNEL‐positive nuclei in the tissue cryopreserved with any of the two methods when compared to that in fresh tissue (Fig. 6).

#### Cultured living myocardial slices

We obtained LMS as described above from fresh and cryopreserved tissue (slow freezing). After 24 h of culture, we fixed the LMS by incubation with 4% PFA at room temperature for 2 h with mechanical agitation and then embedded them in paraffin for sectioning, followed by deparaffinization and IHC to investigate cell composition and morphology (Kruithof et al. 2024; Senesi et al. 2024).

**Figure 7:**
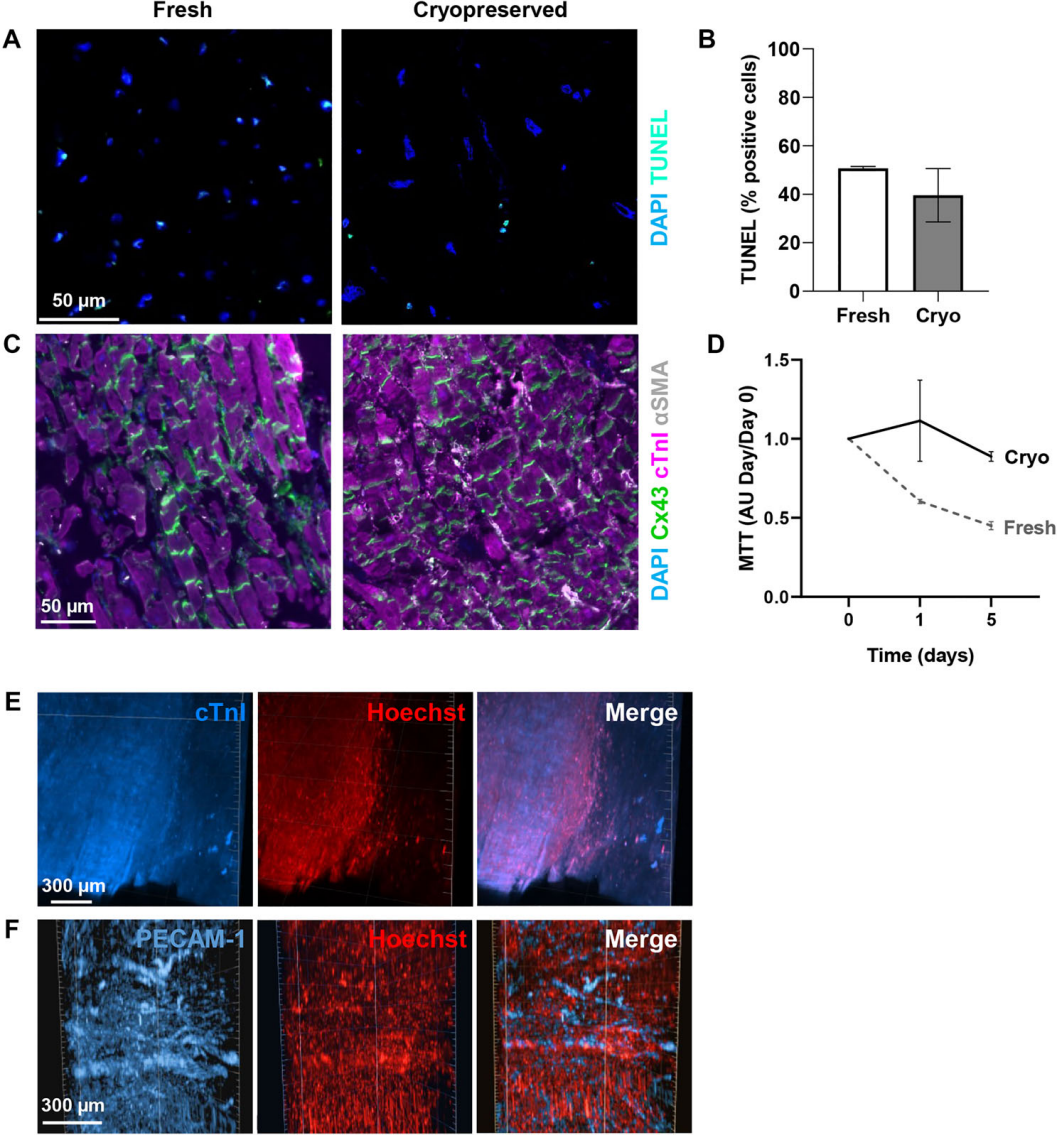
Cultured living myocardial slices (LMS). **(A)** Representative images of apoptotic cells labeled with TUNEL (cyan). Counterstaining of nuclei was performed with DAPI (blue). **(B)** Quantification of apoptotic cells of whole slices labeled with TUNEL from fresh and cryopreserved (Cryo) tissue cultured for 24 h. Values are given as mean ± SD. **(C)** Representative images of immunohistochemical analysis of LMS. Cardiomyocytes were stained with cardiac Troponin‐I antibody (cTnI, magenta), gap junctions between cells with connexin‐43 (Cx43, green), and stromal cell populations with alpha‐smooth muscle actin (αSMA, white). Counterstaining of nuclei was performed with DAPI (blue). **(D)** MTT values in arbitrary units (AU) of LMS from fresh (dashed line) and cryopreserved tissue (Cryo, solid line) on the day of slicing (Day 0) or after culture (Days 1 and 5). Values are given as mean ± SD. **(E)** Representative images showing a 3D lateral view of immunolabeled LMS cultured for 24 h. Cardiomyocytes were stained with cTnI (blue) and nuclei were counterstained with Hoechst (red). **(F)** Representative images showing a 3D lateral view of immunolabeled LMS. Endothelial cells were stained with CD‐31/PECAM‐1 (blue) and nuclei were counterstained with Hoechst (red).

We did not find any significant differences when quantifying markers for apoptosis of whole LMS obtained from cryopreserved tissue compared to fresh tissue (Fig. 7A and B). Additionally, myocardial fiber structure was comparable and Cx43 expression was similarly distributed in LMS from fresh and cryopreserved tissue (Fig. 7C).

We also assessed the viability of cultured LMS from fresh and cryopreserved tissue by determining mitochondrial dehydrogenase activity as previously described (Croft Thomas et al. 2016). Briefly, for each time point, MTT reagent (5 mg/mL thiazolyl blue tetrazolium bromide, Sigma‐Aldrich, cat. no. M5655) was added at 1:10 to the culture medium for 30 min and incubated at 37°C. LMS were then collected and stored at −80°C. After collecting LMS at all time points, the purple formazan precipitate from all the LMS was solubilized in 500 µL of DMSO (dimethylsulfoxide, VWR, cat. no. 1029311000) for 1 h at 37°C. Absorbance in 100 µL was quantified at 550 nm (CLARIOstar Plus plate reader, BMG LABTECH) relative to DMSO alone and referenced with the dry weight of each LMS. Our results suggest that while viability in LMS from fresh tissue tends to decrease with time, viability remains stable in LMS from cryopreserved tissue up to 5 days after slicing, even in the absence of electrical stimulation (Fig. 7D).

To image the full thickness of the myocardial slice, optical clearing methods can be used. We used the iDisco clearing method to immunolabel LMS obtained from cryopreserved tissue and assessed the status of the cardiac fibers and the microvasculature with volume imaging (Renier et al., 2014). We were able to recognize the cardiac fibers and the structure and organization of the microvessels within the LMS by visualizing cTnI‐positive CMs (Fig. 7E) and PECAM‐1‐positive endothelial cells (Fig. 7F), respectively.

Comparable retention of tissue viability and structural integrity post‐cryopreservation (2‐4 weeks of storage) and culture (up to 5 days without electrical stimulation) were observed in cardiac samples and LMS from four different patients.

### Time Considerations

Tissue preparation for cryopreservation according to Basic Protocol 1 requires approximately 15 min, not including reagent preparation. The preparation of LMS according to Basic Protocol 2 may take between 1 and 5 h, depending on the number of slices required. Fixation and staining of tissue sections typically require 20‐40 h for completion, depending on the complexity of the desired staining.

### Author Contributions

**Alessandra M. Lodrini**: Conceptualization; data curation; methodology; writing—original draft; writing—review and editing. **Esmee J. Groeneveld**: Methodology. **Meindert Palmen**: Validation. **Jesper Hjortnaes**: Validation. **Anke M. Smits**: Writing—original draft; writing—review and editing. **Marie‐José** **Goumans**: Data curation; writing—original draft; writing—review and editing.

### Conflicts of Interest

The authors declare no conflicts of interest.

## Data Availability

Data are available on request from the authors.
